# Genome-Wide Identification and Analysis of the Methylation of lncRNAs and Prognostic Implications in the Glioma

**DOI:** 10.3389/fonc.2020.607047

**Published:** 2021-01-08

**Authors:** Yijie He, Lidan Wang, Jing Tang, Zhijie Han

**Affiliations:** ^1^ Department of Bioinformatics, School of Basic Medicine, Chongqing Medical University, Chongqing, China; ^2^ Joint International Research Laboratory of Reproduction & Development, Chongqing Medical University, Chongqing, China

**Keywords:** glioma, methylation modification, long non-coding RNAs, clinical prognosis, the cancer genome atlas (TCGA)

## Abstract

Glioma is characterized by rapid cell proliferation and extensive infiltration among brain tissues, but the molecular pathology has been still poorly understood. Previous studies found that DNA methylation modifications play a key role in contributing to the pathogenesis of glioma. On the other hand, long noncoding RNAs (lncRNAs) has been discovered to be associated with some key tumorigenic processes of glioma. Moreover, genomic methylation can influence expression and functions of lncRNAs, which contributes to the pathogenesis of many complex diseases. However, to date, no systematic study has been performed to detect the methylation of lncRNAs and its influences in glioma on a genome-wide scale. Here, we selected the methylation data, clinical information, expression of lncRNAs, and DNA methylation regulatory proteins of 537 glioma patients from TCGA and TANRIC databases. Then, we performed a differential analysis of lncRNA expression and methylated regions between low-grade glioma (LGG) and glioblastoma multiform (GBM) subjects, respectively. Next, we further identified and verified potential key lncRNAs contributing the pathogenesis of glioma involved in methylation modifications by an annotation and correlation analysis, respectively. In total, 18 such lncRNAs were identified, and 7 of them have been demonstrated to be functionally linked to the pathogenesis of glioma by previous studies. Finally, by the univariate Cox regression, LASSO regression, clinical correlation, and survival analysis, we found that all these 18 lncRNAs are high-risk factors for clinical prognosis of glioma. In summary, this study provided a strategy to explore the influence of lncRNA methylation on glioma, and our findings will be benefit to improve understanding of its pathogenesis.

## Introduction

Glioma is the most common and highly malignant tumor in the intraparenchymal central nervous system (CNS) tumors (1). It is characterized by the rapid and extensive proliferation among brain tissues (2, 3). The high grade glioma subtype, glioblastoma multiform (GBM), could cause the significant mortality that are disproportionate to their relatively rare incidence (4). Even under the best treatment, the median survival time is just over a year, and the few GBM patients survive more than 3 years (1). The etiology and pathogenesis of GBM have been extensively investigated, but the epigenetic mechanisms contributing to its pathogenesis were much less understood (2, 3, 5).

To date, DNA methylation is the most widely studied epigenetic mechanisms (6). Tremendous evidences shows that the DNA methylation is involved in tumorigenesis and development of the GBM (1, 7). For example, the promoter DNA methylation pattern of genes involved in RB1 and TP53 signaling pathways were identified in GBM patients (7). The promoter methylation of the DNA repair enzyme (O6-methylguanine-DNA methyltransferase) was discovered as a significant prognostic factor for temozolomide resistance in GBM patients (8).

The long non-coding RNAs (lncRNAs), a kind of non-protein coding transcripts of >200 nucleotides (9–11), has been reported to be a key regulator in a broad range of biological and cellular processes of GBM, including cell proliferation, motility, hypoxia response, and apoptosis (12–14). The expression levels and functions of lncRNAs could be significantly affected by the genomic methylation in many complex diseases (15–18). Moreover, there is increasing evidences that the methyltransferase, demethylase, and binding protein dynamically regulate the methylation level of the lncRNAs, which influences their expression in specific biological processes (18, 19). However, to date, no systematic study has been conducted to discovery the methylation of lncRNAs and its influences in the glioma on a genome-wide scale.

**Figure 1:**
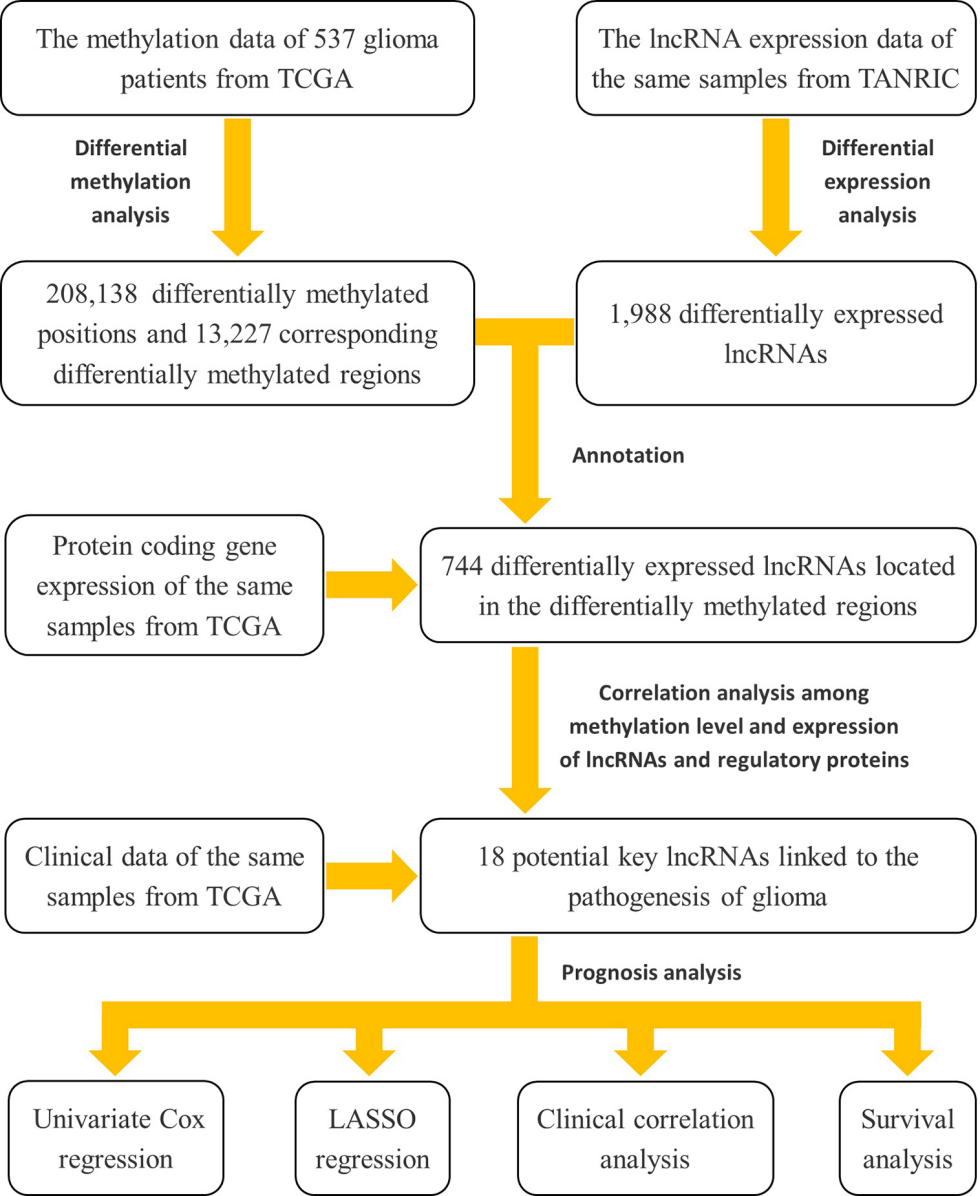
The flow chart of the study design for exploring the potential key lncRNAs contributing the pathogenesis of glioma involved in methylation modifications and their impact on disease prognosis.

Herein, to address this lack of knowledge, we used a cohort of low-grade glioma (LGG) and GBM from The Cancer Genome Atlas (TCGA) database to investigate the contribution of lncRNA methylation to tumorigenesis and development in glioma. Specifically, we first downloaded the expression data of lncRNAs from The Atlas of Noncoding RNAs in Cancer (TANRIC) database, and then implemented a differential expression analysis between the LGG and GBM subjects. Second, we obtained the glioma-related methylation array data and the protein-coding gene expression data of the same samples from TCGA database, and then identified the differentially methylated regions of the differentially expressed lncRNAs according the GENCODE reference annotation for human genomes. Third, we conducted a correlation analysis between methylation level and expression of the lncRNAs and the genes involving in the three kinds of methylation regulatory proteins, and identified the potential key lncRNAs contributing the pathogenesis of glioma. Finally, we conducted the univariate Cox regression, least absolute shrinkage and selection operator (LASSO) regression, clinical correlation, and survival analysis based on the clinical data of these samples to explore the influence of these methylated and potentially disease-related lncRNAs on clinical prognosis of glioma. The flow chart was shown in **Figure 1**.

## Materials and Methods

### Data Collection and Preprocessing

The clinical information and the methylation information of patients with glioma were downloaded from the TCGA database (http://cancergenome.nih.gov), a comprehensive resource for investigating the molecular basis in various cancers. According to TCGA annotation, glioma is classified as the LGG and the GBM. The Genomic Data Commons (GDC) Data Portal (https://portal.gdc.cancer.gov/) was used to access these TCGA data. Particularly, we selected “DNA methylation” in the Data Category, “Illumina human methylation 450” in the Platform, “brain” in the Primary Site, and “gliomas” in the Disease Type to screen out the methylation information of patients. Then, we selected “clinical” in the Data Category, “brain” in the Primary Site, and “gliomas” in the Disease Type to screen out the clinical information of patients. Next, we removed the samples which lack the methylation or clinical information. Finally, the lncRNA expression data of the same patients was downloaded from the TANRIC database, which quantified the expression profiles of lncRNAs in Ensembl using the TCGA data (20).

Moreover, we further searched all the possible studies in PubMed database (http://www.ncbi.nlm.nih.gov/pubmed) using the keywords to “methylase gene,” “methyltransferase gene,” “binding protein gene,” “demethylase gene” to identify the DNA methylation regulatory proteins. The search was performed before the last update of this database on May 13, 2020. The gene expression of the methylation regulatory proteins was obtained from TCGA database. The expression data of lncRNAs and methylation regulatory proteins have been normalized as reads per kilobase of exon model per million mapped reads (RPKM) and RNA-Seq by Expectation-Maximization (RSEM), respectively. The DNA methylation values were normalized using the “betaqn” function of the R package “wateRmelon” (http://bioconductor.org/packages/release/bioc/html/wateRmelon.html) (21).

### Differential Expression Analysis of lncRNAs Between Low-Grade Glioma and Glioblastoma Multiform

To identify the key lncRNAs which are potentially associated with the gliomas progression, we performed a differential expression analysis of all the lncRNAs obtained from TANRIC database between LGG and GBM subjects using the R package “lncDIFF” with its default parameter settings (i.e. link.function = “log,” simulated.pvalue = FALSE, permutation = 100) (https://CRAN.R-project.org/package=lncDIFF). It is a powerful differential analysis tool by the normalized expression data (e.g. RPKM values) as the input, and has high sensitivity to identify the low abundant differentially expressed genes, as commonly observed in lncRNAs. This package adopts the generalized linear model with zero-inflated exponential quasi-likelihood to estimate group effect on normalized counts, and employs the likelihood ratio test to detect differential expressed genes. The proposed method and tool are applicable to data processed with standard RNA-seq preprocessing and normalization pipelines (22). We first removed 21 lncRNAs whose expression is zero in all the LGG or GBM subjects. Then, we set significance level according to the common threshold of the absolute value of fold change (*FC*) ≥ 2 and false discovery rate (FDR) *p* < 0.05. The *p* values are corrected for multiple testing by Benjamini–Hochberg method. Finally, we used a volcano plot to describe the profile of whole lncRNA expression by the R package “ggplot2” (https://CRAN.R-project.org/package=ggplot2), and used a heatmap to visualize the cluster pattern of the differentially expressed lncRNAs based on Manhattan distance by the R package “gplots” (https://CRAN.R-project.org/package=gplots).

### Differential Methylation Analysis and lncRNA Annotation

To identify the glioma-related methylation positions and regions, and the differentially expressed lncRNAs located in these regions, we performed a differential methylation analysis and lncRNA annotation. The differential methylation analysis was conducted by the R package “minfi” which is a specialized tool designed to process the Illumina methylation 450 array data (http://bioconductor.org/packages/release/bioc/html/minfi.html). It used the Subset-quantile Within Array Normalization method to preprocess data and the bump-hunting algorithm to discover the differential methylation information (23). Firstly, we used the “densityBeanPlot” function of this package to conduct the quality control for each array. The qualified samples should have the characteristic that the methylation levels (beta values) of CpG positions are distributed around 0 and 1, respectively. Then, we used the “dmpFinder” function (type = “categorical”) of this package to identify the differentially methylated positions between LGG and GBM subjects based on the methylation array. The significance level was set according to a common threshold of the absolute intercept ≥ 0.2 (i.e. 20% difference on the beta values) and *p* < 1×10^−3^ (24). Next, based on these differentially methylated positions, we further used the “bumphunter” function (B = 10, type = “Beta”) of this package to look for the differentially methylated regions between LGG and GBM subjects with the common threshold of average methylation level difference ≥ 0.2 (25, 26). The differentially methylated regions are the consecutive genomic locations containing a battery of differentially methylated positions in the same direction. Finally, we download the ensGene annotation file (hg19) from the Ensembl (release 75) which stores the location information of lncRNA transcripts and exons in human genome. Based on this file, the ANNOVAR software was used to perform an lncRNA annotation and identify the differentially expressed lncRNAs located in the differentially methylated regions. ANNOVAR is a Perl command-line tool for rapidly and efficiently annotating the genomic variants, including gene-based, region-based and filter-based annotations on a variant call format (VCF) file generated from human genomes (27).

### Correlation Analysis Between Methylation and Expression of lncRNAs

To explore the influence of methylation on the corresponding lncRNAs, and identify the potential key lncRNAs contributing the pathogenesis of glioma, we performed a correlation analysis between methylation and expression of lncRNAs. Particularly, we first selected the differentially methylated positions to be included in each of the identified lncRNAs in the previous step, and calculated the average values of these methylation positions for each lncRNAs, respectively. Then, we calculated the Pearson’s correlation coefficient between the expression of these lncRNAs and their average methylation level using the R function “cor.test.” The threshold of significance was set at the absolute value of r > 0.6 and FDR p < 0.05. The p values are corrected for multiple testing by Benjamini–Hochberg method. Finally, it is reported that the methylation regulatory proteins (including methyltransferase, demethylase, and binding protein) dynamically regulate the methylation level of lncRNAs, which influences their expression in specific biological processes (18, 19). Therefore, to explore which methylation regulatory proteins are involved in the methylation modification of the potential key lncRNAs and further increase the reliability of our findings, we selected the known methylation regulatory proteins and calculated the Pearson’s correlation coefficient between their expression and the average methylation level of these lncRNAs using the same significance threshold.

### Influence of the Methylated lncRNAs on Clinical Prognosis of Glioma

We further analyzed the Influence of these identified methylated lncRNAs potentially contributing the pathogenesis of glioma on the clinical prognosis of glioma. First, we calculated the average expression of the key lncRNAs obtained above in each patient and get the median of these average expressions. According to the median, the patients were separated into the lncRNAs low and high expression groups. We compared prognosis between the high expression and low expression subjects using a Kaplan-Meier overall survival curves. Then, we performed a univariate Cox regression analysis to assess the association between these methylated lncRNAs and the prognosis of glioma. The threshold of significance was set at 95% confidence interval (CI) of hazard ratio (HR) ⊉ 1 and *p* < 0.05. The R package “survival” (https://CRAN.R-project.org/package=survival) was used for these analyses. Next, based on the results of univariate Cox regression analysis, we further used the LASSO regression algorithm to identify the key lncRNAs whose methylation and expression impact on the prognosis of glioma by R package “glmnet.” It is a pathwise algorithm for the Cox proportional hazards model, regularized by convex combinations of ℓ1 and ℓ2 penalties (elastic net). The algorithm fits *via* cyclical coordinate descent, and employs warm starts to find a solution along a regularization path (28). The parameter familiy, maxit, and alpha were set to Cox, 1000 and 1, respectively (others were set by their default values). And then we calculated the risk score of each subject using them through the “survival” package. Finally, we used a receiver operator characteristic (ROC) curve to verify the reliability of the risk score by the R package “survivalROC” (https://CRAN.R-project.org/package=survivalROC). In addition, we also assessed the association between these lncRNA expressions and other clinical features of the patients (including age at initial pathologic diagnosis, vital status, and gender) using the chi-square test. The threshold of significance was set at *p* < 0.05.

## Results and Discussion

### Methylation, Expression, and Clinical Information of 537 Glioma Samples

After the data collection, we found a total of 537 glioma samples (including 486 LGG and 51 GBM patients from TCGA) with the DNA methylation values, expression levels of protein-coding genes, and clinical information. Particularly, according to the annotation of Illumina human methylation 450 array, a total of 369,531 CpGs methylation positions were quantified after removing the missing values. We normalized the CpGs methylation values for the subsequent analyses. The results were shown in **Supplementary Figure S1**. The lncRNA expression data of the 537 glioma samples were obtained from TANRIC database. A total of 12,727 lncRNAs of these samples were quantified as RPKM values. Through the keyword search and the title/abstract screening, 23 articles containing genes for methylation-related enzymes were obtained from PubMed. In total, we identified 32 DNA methylation regulatory proteins (including methyltransferase, demethylase, and binding protein) from the 23 articles (**Table 1**). We extracted the expression data of these 32 methylation regulatory proteins for each sample (quantified as RSEM values) from the TCGA database. The clinical information of these samples contains age, gender, survival time, and vital status. The summary of these glioma samples was listed in **Table 2**.

**Table 1:** The information of the 32 DNA methylation regulatory proteins.

Gene	ID	Description	Type	Reference
DNMT3A	1788	DNA methyltransferase 3 alpha	Methyltransferase	(29, 30, 31)
DNMT3B	1789	DNA methyltransferase 3 beta	Methyltransferase	(29, 30, 31)
DNMT3L	29947	DNA methyltransferase 3 like	Methyltransferase	(30)
DNMT1	1786	DNA methyltransferase 1	Methyltransferase	(31–33)
DMAP1	55929	DNA methyltransferase 1 associated protein 1	Binding protein	(33)
SUV39H1	6839	Suppressor of variegation 3-9 homolog 1	Methyltransferase	(34)
MECP2	4204	Methyl-CpG binding protein 2	Binding protein	(35)
MBD1	4152	Methyl-CpG binding domain protein 1	Binding protein	(35)
MBD2	8932	Methyl-CpG binding domain protein 2	Binding protein	(35)
MBD3	53615	Methyl-CpG binding domain protein 3	Binding protein	(35)
MBD4	8930	Methyl-CpG binding domain 4, DNA glycosylase	Binding protein	(35)
SETDB1	9869	SET domain bifurcated histone lysine methyltransferase 1	Methyltransferase	(31, 35)
MGMT	4255	O-6-methylguanine-DNA methyltransferase	Methyltransferase	(36)
TET1	80312	tet methylcytosine dioxygenase 1	Demethylase	(37)
TET2	54790	Tet methylcytosine dioxygenase 2	Demethylase	(37)
TET3	200424	Tet methylcytosine dioxygenase 3	Demethylase	(37)
JMJD6	23210	Jumonji domain containing 6, arginine demethylase and lysine hydroxylase	Demethylase	(38)
KDM3A	55818	Lysine demethylase 3a	Demethylase	(39)
KDM5C	8242	Lysine demethylase 5c	Demethylase	(39)
KDM1A	23028	Lysine demethylase 1a	Demethylase	(40)
KDM5B	10765	Lysine demethylase 5b	Demethylase	(41)
KDM5A	5927	Lysine demethylase 5a	Demethylase	(42)
KDM5D	8284	Lysine demethylase 5d	Demethylase	(42)
KDM3B	51780	Lysine demethylase 3b	Demethylase	(43)
KDM4A	9682	Lysine demethylase 4a	Demethylase	(44, 45)
KDM4B	23030	Lysine demethylase 4b	Demethylase	(46)
KDM4C	23081	Lysine demethylase 4c	Demethylase	(47)
KDM4D	55693	Lysine demethylase 4d	Demethylase	(48)
KDM6A	7403	Lysine demethylase 6a	Demethylase	(42, 49)
KDM6B	23135	Lysine demethylase 6b	Demethylase	(42, 49)
KDM2A	22992	Lysine demethylase 2a	Demethylase	(48)
KDM2B	84678	Lysine demethylase 2b	Demethylase	(50, 51)

**Table 2:** Summary of the 537 individuals studied in this work.

Individuals	Sample Type	Sample Size	Mean Age (SD)	Male/Female (%)	Death Rates (%)
GBM subjects	Primary Tumor	51	61.54 (13.41)	56.00/44.00	66.00
LGG subjects	Primary Tumor	486	42.91 (13.42)	54.64/45.36	25.15
Total		537	44.66 (14.48)	54.77/45.23	28.97

### Differential Expression Analysis of lncRNAs Between Low-Grade Glioma and Glioblastoma Multiforme

**Figure 2:**
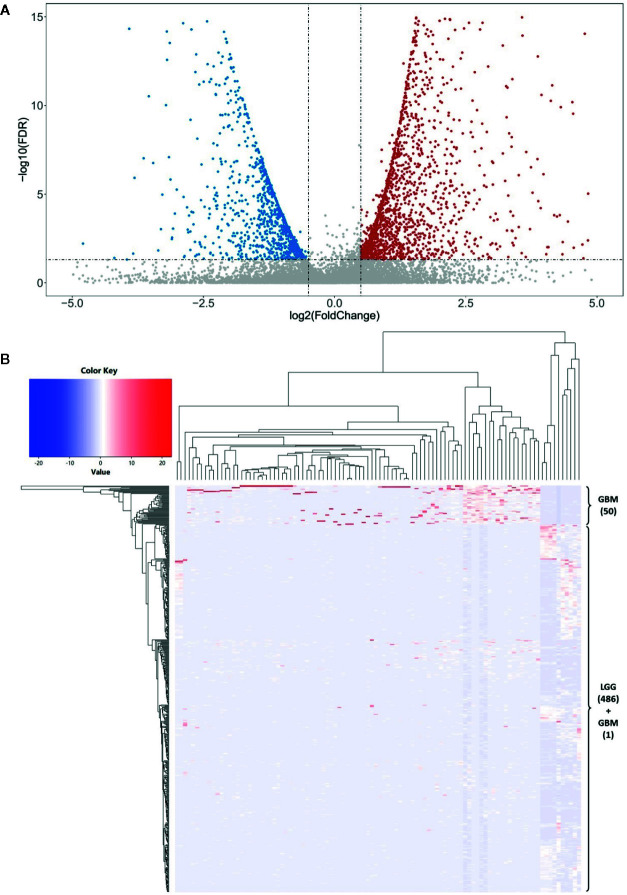
The differential expression analysis of lncRNAs. **(A)** The volcano plot shows a profile of the expression of all these lncRNAs in this study. There are 1,284 highly expressed (i.e. and FDR *p* < 0.05) and 704 lowly expressed lncRNAs (and FDR *p* < 0.05) in the GBM subjects. **(B)** The clustered heatmap of the top 100 most differentially expressed lncRNAs between LGG and GBM subjects. Most of these lncRNAs (83%) are significantly highly expressed in the GBM than LGG subjects. There are 50 GBM subjects (about 98.04%) grouped into a cluster base on the lncRNA expression.

We used the R package “lncDIFF” to perform the differential expression analysis of lncRNAs between LGG and GBM subjects according to the significance threshold of |*FC*| ≥ 2 and FDR *p* < 0.05. In total, we identified 1,988 significantly differentially expressed lncRNAs, which include 1,284 highly expressed (i.e. *FC* ≥ 2) and 704 lowly expressed lncRNAs (*FC* ≤ −2) in the GBM subjects. The details are described in **Supplementary Table S1**. We used a volcano plot to describe the profile of whole lncRNA expression (**Figure 2A**). Then, to verify these findings, we contrasted these identified differentially expressed lncRNAs with another independent study. This study used 19 glioblastoma and 9 control brain samples to perform the differential expression analysis of 30,586 lncRNA transcripts (Arraystar Human lncRNA Microarray V3.0, nearly 30% of them overlap with our study). According to its results, about 71.5% differentially expressed lncRNAs overlapped with our findings (52). Further, we selected the top most 100 differentially expressed lncRNAs to visualize the cluster pattern of their expression by a heatmap. As **Figure 2B** shows, the GBM and LGG subjects are mainly grouped under a cluster according to high and low expression of the lncRNAs, respectively, and most of these lncRNAs (83%) are significantly highly expressed in the GBM than LGG subjects. These differentially expressed lncRNAs may contribute to the progress of glioma. Thus, we used these significantly differentially expressed lncRNAs to conduct the subsequent analysis.

### Differential Methylation Analysis and lncRNA Annotation

We performed a differential methylation analysis and lncRNA annotation to identify the glioma-related DNA methylation and the differentially expressed lncRNAs located in the regions. The results of array quality control showed that the beta values of DNA methylation positions are mainly distributed around 0 and 1, respectively, for each sample (**Supplementary Figure S2**). Then, we used these arrays to identify the differentially methylated positions and regions, respectively. The results showed that there are a total of 208,138 positions and 13,227 corresponding regions with a significantly differential methylation level between LGG and GBM subjects (**Supplementary Tables S2**, **S3**). The methylation array GSE90496 (contains 347 GBM and 301 LGG subjusts) was used to verify these findings. The results showed that about 71.1% differential methylation positions are consistent with our findings (53). Finally, based on these differentially methylated regions, we performed the location annotation of the differentially expressed lncRNAs. In total, we identified 744 lncRNAs which are located in the differentially methylated regions. According to the results of annotation, these differentially methylated regions are at five categories of different genomic locations, i.e. intergenic, ncRNA_exonic, ncRNA_intronic, upstream and downstream, and the proportion of intergenic areas is significantly increased compared with other types (**Supplementary Table S4**). We further calculated the proportion of them in the different genomic locations. We found that these lncRNAs are mainly distributed in chromosome 1, 2, 7, and 12 (11.42, 10.48, 8.06, and 8.06%, respectively) (**Supplementary Table S5**). Moreover, as the **Supplementary Table S1** shown, these identified lncRNAs include 1,284 highly and 704 lowly expressed ones in the GBM subjects. But not all of these highly and lowly expressed lncRNAs have significantly reduced and increased methylation levels in the GBM subjects, respectively, which imply that not all of DNA methylation changes can affect the expression of lncRNAs in the corresponding genomic regions.

### Correlation Analysis Between Methylation and Expression of lncRNAs

We first conducted a Shapiro-Wilk normality test for each vector by the R function “shapiro.test.” According to the threshold *P* > 0.05, 11 lncRNAs that do not obey the normal distribution were removed. Then, to identify the differentially expressed lncRNAs affected by the glioma-related DNA methylation, we performed a Pearson’s correlation analysis between methylation and expression of lncRNAs. The results revealed that there are a total of 18 lncRNAs (including 16 highly and 2 lowly expressed ones) whose expression is significantly associated with their DNA methylation level, and all of them show a significant negative correlation (*r* < −0.6 and FDR *p* < 0.05) (**Table 3**). It is consistent with the common understanding that DNA methylation inhibits the corresponding gene expression in a variety of tissues and cell lines (54, 55). Next, we further measured the association between the expression of the 32 methylation regulatory proteins and methylation level of these lncRNAs. The results showed that there is a significantly negative correlation between *TET1* expression and most of the 18 lncRNAs’ methylation level. *TET1* is a demethylase which can catalyze the conversion of 5-methylcytosine to 5-hydroxymethylcytosine and maintains hypomethylation status of the corresponding regions (37). Besides this gene, the expression of *KDM4B* and *MBD2* also show a significantly negative and positive correlation (*r* > 0.6 and FDR *p* < 0.05) with a part of the 18 lncRNAs’ methylation level, respectively. *KDM4B* is also a demethylase of histone lysine by a hypoxia-induced pathway, and an important epigenetic modifier in cancer (46). *MBD2* is a methyl-CpG binding protein which binds and maintains methylated gene promoter to repress its transcriptional activity (35). However, this significant correlation is not observed in the other 29 genes, which imply that the DNA methylation of lncRNAs in glioma may be influenced predominantly by some specific methylation regulatory proteins (**Table 3** and **Supplementary Table S5**). Moreover, the mean differential methylation of the 18 lncRNAs was calculated. We found that all of the lowly expressed lncRNAs have significantly reduced methylation levels and most of them have significantly reduced methylation levels in the GBM subjects. It is also consistent with the understanding that DNA methylation inhibits gene expression (56, 57). Therefore, we considered them as the potential key lncRNAs contributing the pathogenesis of glioma involved in methylation modifications. Finally, we further queried PubMed for the functions of the 18 potential key lncRNAs contributing the pathogenesis of glioma. We found that seven of them have been demonstrated to be functionally linked to the pathogenesis of glioma by the previous studies. For example, the overexpression of the ENSG00000222041 (CYTOR) partially reversed the inhibitory effects of UPF1 on proliferation and invasion abilities in glioma (58, 59). The more details were described in the **Supplementary Table S6**.

**Table 3:** The information of the 16 potential key lncRNAs contributing the pathogenesis of glioma involved in methylation modifications.

LncRNAs	Symbol	Differentially Methylated Regions			Fold Change (FDR)	Correlation LL (FDR)	Correlation LP (FDR)			MeanDM
		Chr	Start	End			TET1	KDM4B	MBD2	
ENSG00000177738	AC025171.1	chr5	43312201	43312201	2.09 (5.1E-07)	−0.63 (4.8E-60)	−0.71 (6.5E-82)	−0.61 (1.4E-54)	0.52 (2.4E-37)	−1.985
ENSG00000222041	CYTOR	chr2	88751786	88752553	7.32 (7.3E-65)	−0.62 (4.4E-57)	−0.72 (2.8E-85)	−0.63 (1.6E-60)	0.61 (1.7E-54)	−0.800
ENSG00000224081	SLC44A3-AS1	chr1	95286125	95286125	2.85 (1.5E-14)	−0.65 (1.0E-64)	−0.66 (1.1E-66)	−0.60 (7.4E-53)	0.51 (1.2E-35)	0.801
ENSG00000226445	BX322234.1	chr6	169613210	169613372	2.15 (8.4E-08)	−0.64 (4.2E-62)	−0.67 (6.7E-70)	−0.61 (5.3E-54)	0.57 (2.5E-47)	2.763
ENSG00000227372	TP73-AS1	chr1	3807168	3807312	2.81 (2.6E-14)	−0.90 (2.2E-189)	−0.72 (3.2E-84)	−0.62 (4.8E-58)	0.50 (6.7E-34)	−2.153
ENSG00000230074	AL162231.2	chr9	34666173	34666173	3.89 (1.6E-25)	−0.79 (4.3E-112)	−0.72 (3.2E-84)	−0.63 (3.2E-59)	0.50 (2.9E-34)	−2.442
ENSG00000231609	AC007098.1	chr2	63850056	63850056	2.94 (6.2E-11)	−0.65 (3.5E-64)	−0.66 (1.2E-65)	−0.53 (8.2E-39)	0.50 (3.5E-35)	−0.246
ENSG00000232533	AC093673.1	chr7	143081287	143081287	3.73 (1.5E-24)	−0.78 (1.3E-108)	−0.63 (3.0E-58)	−0.48 (2.2E-31)	0.57 (3.7E-46)	−1.423
ENSG00000234883	MIR155HG	chr21	26934424	26934885	6.02 (1.2E-50)	−0.61 (1.9E-54)	−0.74 (2.9E-90)	−0.62 (1.2E-56)	0.54 (2.9E-40)	−0.780
ENSG00000246100	LINC00900	chr11	115631116	115631389	2.96 (7.0E-16)	−0.71 (3.1E-80)	−0.68 (1.7E-73)	−0.57 (1.7E-46)	0.49 (5.4E-33)	−2.839
ENSG00000249249	AC010226.1	chr5	114937919	114938439	3.75 (8.7E-25)	−0.74 (5.8E-92)	−0.75 (2.0E-96)	−0.65 (6.4E-65)	0.58 (6.4E-48)	−2.047
ENSG00000249859	PVT1	chr8	129284837	129284837	4.31 (3.6E-31)	−0.72 (2.1E-84)	−0.68 (1.1E-73)	−0.56 (1.1E-44)	0.60 (1.8E-53)	1.356
ENSG00000250786	SNHG18	chr5	9546404	9546404	3.55 (1.6E-22)	−0.66 (1.0E-65)	−0.74 (4.5E-90)	−0.63 (1.6E-60)	0.53 (8.7E-39)	−2.027
ENSG00000251131	AC025171.3	chr5	43019660	43020716	2.59 (7.6E-12)	−0.65 (1.9E-64)	−0.72 (1.8E-86)	−0.64 (2.4E-61)	0.51 (1.6E-36)	−2.032
ENSG00000254675	AP003032.1	chr11	77734334	77734334	3.85 (1.4E-02)	−0.61 (2.4E-55)	−0.71 (4.3E-83)	−0.60 (2.9E-53)	0.55 (1.9E-42)	−0.167
ENSG00000255571	MIR9-3HG	chr15	89921845	89922989	-2.99 (2.3E-09)	−0.65 (8.0E-64)	−0.13 (3.9E-02)	−0.13 (3.9E-02)	−0.14 (3.9E-02)	0.215
ENSG00000256802	AC022613.1	chr15	29969096	29969096	3.71 (2.6E-24)	−0.62 (1.0E-56)	−0.68 (8.6E-72)	−0.59 (1.8E-51)	0.54 (1.4E-41)	−1.557
ENSG00000263874	LINC00672	chr17	37081875	37081875	-2.72 (4.6E-08)	−0.75 (1.1E-95)	0.004 (9.9E-01)	0.02 (9.3E-01)	0.03 (9.3E-01)	0.138

Correlation LL, correlation between the average methylation level of lncRNAs and their expression; Correlation LP, correlation between the methylation level of lncRNAs and the expression of methylation regulatory proteins; MeanDM, mean differential methylation level.

### Influence of the Methylated lncRNAs on Clinical Prognosis of Glioma

**Figure 3:**
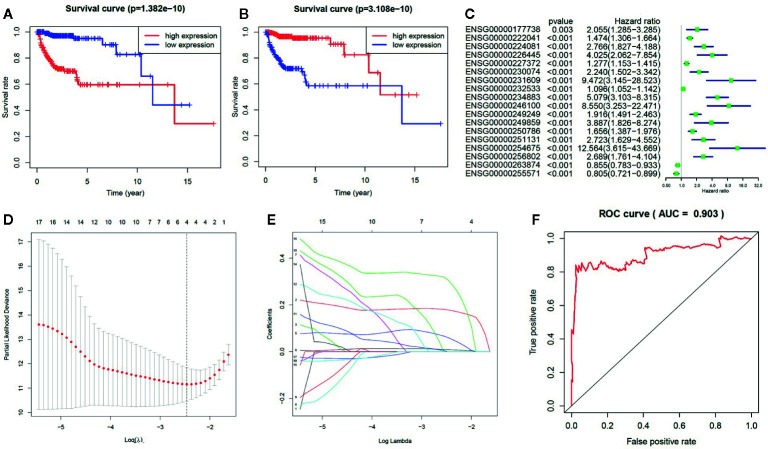
The influence of the methylated lncRNAs on the prognosis of glioma. **(A)** For the 16 lncRNAs highly expressed in GBM patients, the Kaplan-Meier overall survival curve of the subjects with high lncRNA expression is significantly longer than the subjects with low lncRNA expression. **(B)** For the two lncRNAs lowly expressed in GBM patients, the Kaplan-Meier overall survival curve of the subjects with low lncRNA expression is significantly longer than the subjects with high lncRNA expression. **(C)** The forest plot for the results of univariate Cox regression analysis in LGG. **(D)** The relationship between the partial likelihood deviance and the penalty coefficient λ value. The log (λ) is equal to about 2.5 when the partial likelihood deviance reaches its minimum value. **(E)** The LASSO regression for calculating the coefficient of each lncRNA. There are four lncRNAs with non-zero coefficients when the log (λ) is equal to 2.5. **(F)** The ROC curve shows the reliability of the risk score.

**Figure 4:**
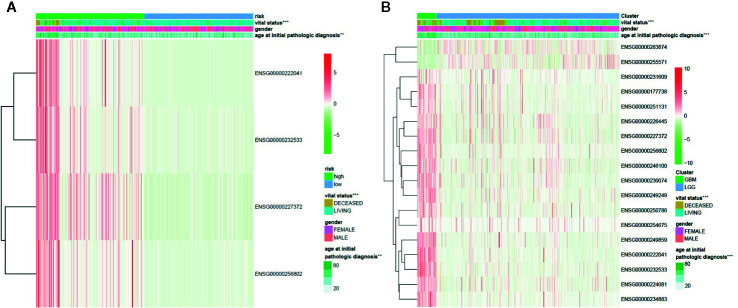
The association between the methylated lncRNAs and the clinical features of glioma patients. **(A)** The risk classification by the four key methylated lncRNAs is significantly associated with the age at initial pathologic diagnosis and vital status, but not with the gender of the patients. **(B)** The 18 glioma-related lncRNAs are significantly differentially expressed between GBM and LGG groups, which are also significantly associated with the age and vital status, but not with the gender.

We further analyzed the influence of the 18 identified lncRNAs, which potentially contribute the glioma pathogenesis by methylation modifications, on the clinical prognosis of glioma. For the 16 lncRNAs highly expressed in GBM patients, we found that the overall survival curve of the subjects with high lncRNA expression is significantly longer than the subjects with low lncRNA expression (*p* = 1.38 × 10^−10^) (**Figure 3A**). On the contrary, the overall survival curve of the subjects with low lncRNA expression is significantly longer than the subjects with high lncRNA expression for the two lowly expressed lncRNAs (*p* = 3.11 × 10^−10^) (**Figure 3B**). It reflects an association between the dysregulation of lncRNA expression and a bad prognosis of glioma patients. To avoid dependence on the tumor grade, we performed the univariate Cox regression analysis of the 18 lncRNAs in GBM and LGG subjects, respectively. We did not find a significant association between lncRNA expression and poor patient outcomes in GBM subjects. However, the results showed that all of the 18 identified methylated lncRNAs are high-risk factors for the prognosis of glioma in LGG subjects (i.e. 95% CI HR ⊉ 1 and *p* < 0.001) (**Figure 3C**). This suggests that both over-expression of those 16 lncRNAs and under-expression of other 2 ones can lead to a poor prognosis in LGG patients, which is also consistent with common sense, given that GBM patients are in advanced stages of the disease and their survival may be affected by other complications or factors. In addition, the univariate Cox regression analysis was further performed for all the 1,988 significantly differentially expressed lncRNAs in LGG patients. We found that only about 39% of the lncRNAs unlikely affected by methylation modifications are associated with poor patient outcomes (**Supplementary Table S7**). We further applied LASSO regression algorithm to the 18 lncRNAs to identify the key ones for glioma prognosis and calculate the risk score of each subject. As **Figures 3D, 3E** show, there are four key lncRNAs (i.e. ENSG00000256802, ENSG00000232533, ENSG00000227372, ENSG00000222041) selected when the cross-validated partial likelihood deviance reaches its minimum value, and the coefficients of all these lncRNAs are positive (i.e. increase risk of disease). The area under the curve (AUC) of the ROC is 0.903, which shows the reliability of the risk score (**Figure 3F**). According to the median of risk scores, the patients were separated into the low and high-risk groups. We found that the GBM subjects are mainly distributed in high-risk group, while the LGG subjects are mainly distributed in low-risk group. This demonstrates the consistency between the sample risk score by the key lncRNAs and the severity of glioma. Moreover, as **Figure 4A** shows, the risk classification by the key lncRNAs is significantly associated with the age at initial pathologic diagnosis (*p* = 1.32 × 10^−2^) and vital status (*p* = 1.72 × 10^−8^). But we observed no association with the gender of the patients (*p* = 1.97 × 10^−1^). The similar results were also observed for all the 18 identified methylated lncRNAs (*p* value of age, vital status and gender is 2.87 × 10^−14^, 2.01 × 10^−9^, and 1.45 × 10^−1^, respectively) (**Figure 4B**).

## Conclusions

In this study, we used the TCGA data to identify potential key lncRNAs contributing the pathogenesis of glioma involved in methylation modifications and further explore influence of them on the clinical prognosis of glioma. In total, we identified 18 such lncRNAs which has the following four characteristics: 1) they are significantly differentially expressed between the LGG and GBM subjects; 2) at least one of the differentially methylated regions, which cover the contiguous differentially methylated positions, is located in these lncRNA sequences; 3) there is a strong correlation between the methylation level of these lncRNAs and the expression of methylation regulatory proteins; 4) the expression of these lncRNAs is significantly associated with their methylation level. Further, the results of clinical data analysis show that all these 18 lncRNAs are high-risk factors for the clinical prognosis of glioma, and four of them (i.e. ENSG00000256802, ENSG00000232533, ENSG00000227372 and ENSG00000222041) are most important for the severity of glioma. All in all, we performed a strategy to explore the influence of the lncRNA methylation on the pathogenesis of glioma, and these findings will be benefit to further glioma research in the future.

## Data Availability Statement

Publicly available datasets were analyzed in this study. This data can be found here: TCGA (http://cancergenome.nih.gov), GDC Data Portal (https://portal.gdc.cancer.gov/), and PubMed (http://www.ncbi.nlm.nih.gov/pubmed).

## Author Contributions

ZH designed the research. ZH, YH, JT, and LW collected the data. ZH and YH performed the research and analyzed data. ZH, JT, and YH wrote the paper. ZH and JT reviewed and modified the manuscript. All authors discussed the results and contributed to the final manuscript. All authors contributed to the article and approved the submitted version.

## Funding

This research is financially supported by the Start-up fund of Chongqing Medical University (R1017).

## Conflict of Interest

The authors declare that the research was conducted in the absence of any commercial or financial relationships that could be construed as a potential conflict of interest.
