# Supplementary material for: Genome-Wide Identification and Analysis of the Methylation of lncRNAs and Prognostic Implications in the Glioma
Source: Front Oncol. 2021 Jan 8;10:607047. doi: 10.3389/fonc.2020.607047 (PMC7820673; doi:10.3389/fonc.2020.607047)
Supplement: Supplementary file 1 [file DataSheet_1.docx]

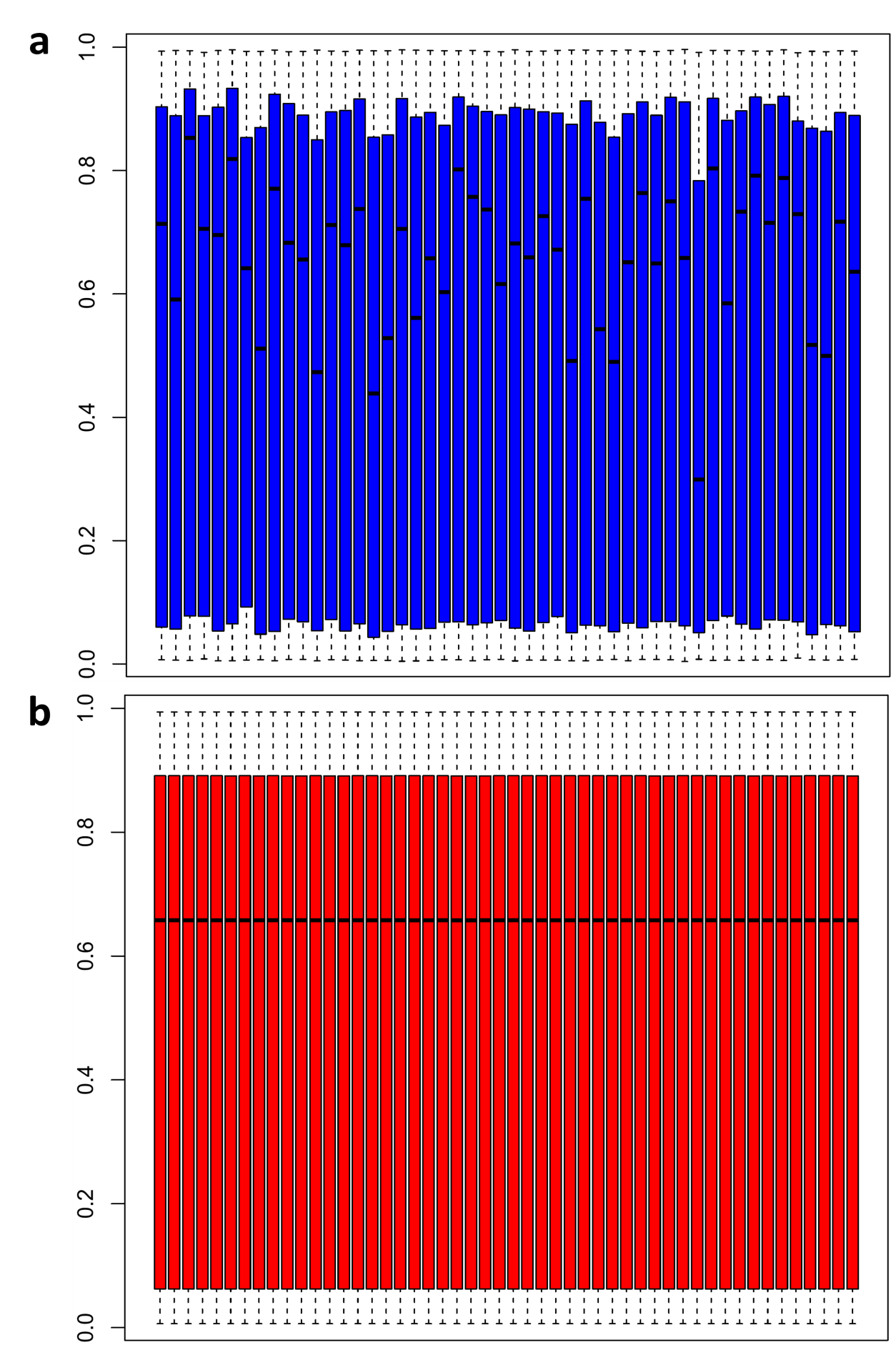


**Figure S1** The results of the DNA methylation microarray data normalization. **(a)** The boxplot reveals the distribution of methylation values in 50 glioma samples selected at random without normalization processing. **(b)** The boxplot reveals the distribution of methylation values in the 50 samples after the normalization processing.


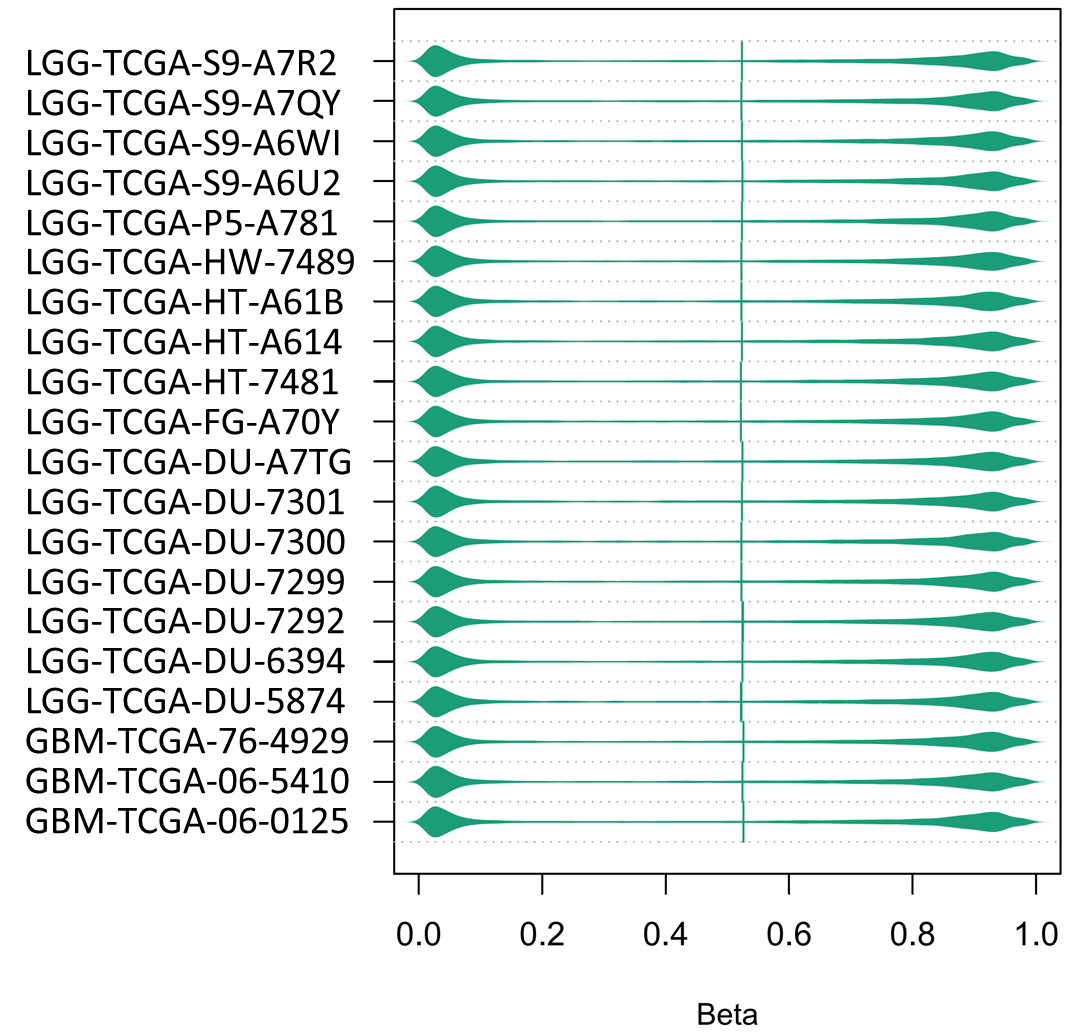


**Figure S2** The results of the DNA methylation array quality control. The density bean plot reveals the distribution of the beta values of CpG methylation sites in 20 glioma samples (i.e. methylation array) selected at random.
